# Sensitization to inhaled and food allergen sources in patients with allergic diseases in Eastern China

**DOI:** 10.3389/falgy.2025.1616730

**Published:** 2025-08-20

**Authors:** Zhibang Hu, Chunhui Wang, Jianrong Xue, Shiyu Yang, Yongzheng Bao, Yunhui Wu, Xiaoju Hou, Yishake Kaiseer, Jing Ma

**Affiliations:** ^1^ Department of Otorhinolaryngology, Changzhou Third People’s Hospital, Changzhou Medical Center, Nanjing Medical University, Changzhou, China; ^2^ Department of Eye and ENT, The People’s Hospital of Wuqia, Kezhou, China

**Keywords:** allergen sources, inhaled, food, allergic diseases, specific IgE, Eastern China

## Abstract

**Background:**

Allergen exposure plays a critical role in the onset of allergic disease, and the distribution of allergens varies by geographic location, climate, and lifestyle.

**Methods:**

A retrospective analysis was conducted on the data of 4,149 patients with clinically suspected allergic diseases who sought medical care at Changzhou Third People's Hospital. The total IgE and specific IgE (sIgE) levels for 19 inhaled and food allergen sources were assessed using the Mediwiss AllergyScreen system. The sensitization patterns to various allergen sources were delineated based on sex and age categories.

**Results:**

A total of 2,491 (60.04%) patients were positive for sIgE to at least one allergen source. Specifically, 997 (40.02%) patients were positive for one allergen source, while 1,494 (59.98%) were positive for two or more allergen sources. The sensitization rate for inhaled allergen sources was 49.41% (2,050), which was significantly higher than that for food allergen sources at 36.61% (1,519) (*p* < 0.05). The most commonly inhaled allergen source was *D. pteronyssinus* (37.58%, 1,559/4,149), followed by mold mix (13.11%, 544/4,149) and house dust (9.11%, 378/4,149). Among food allergen sources, the most prevalent reactions were cashew nut (15.57%, 646/4,149), cow's milk (10.99%, 456/4,149), and egg (9.33%, 387/4,149). The majority of sIgE levels were concentrated in the lower grades (grade I to III), while eight cases of crab and six cases of shrimp allergen sources presented at the highest responses (grade IV). Males exhibited significantly higher sIgE-positive rates for both inhaled and food allergen sources than females (*p* < 0.05). Additionally, the sIgE-positive rates for most inhaled and food allergen sources displayed significant variations across different age groups (*p* < 0.05). The highest sIgE-positive rate for inhaled allergen sources was observed in the 11–15 years group, and the positive rates of sIgE for food allergen sources decreased with advancing age. The levels of total serum IgE also varied among different age groups, with 2,631 (63.41%) individuals belonging to the high-concentration group. Total serum IgE exhibited an upward trend until the age of 11–15 years, which was then followed by a steady decrease.

**Conclusion:**

The results revealed the sensitization characteristics of allergen sources in eastern China, providing valuable insights into the prevention, diagnosis, and management of allergic diseases in this region.

## 1 Introduction

Allergic diseases are a group of chronic inflammatory disorders that occur after exposure to allergens, including allergic rhinitis (AR), asthma, atopic dermatitis, and drug and food allergy. In some severe cases, patients may even progress to anaphylaxis (1). The symptoms of allergic diseases are diverse, mainly including nasal itching, sneezing, runny nose, red or itchy eyes, tearing, skin itching, coughing, shortness of breath or breathlessness, abdominal pain, diarrhea, and vomiting. These symptoms significantly impair patients’ quality of life and can even threaten their lives (2–4). Over the past few decades, industrial development and lifestyle changes have led to varying rates of increase in the prevalence of allergic diseases (5, 6). Indeed, it is estimated that approximately 30%–40% of the global population suffers from one or more allergic conditions, thereby imposing a significant medical and economic burden on those affected (7, 8).

The pathogenesis of allergic diseases remains unclear, with both genetic and environmental factors playing critical roles in their occurrence and progression (9). The primary trigger for allergic diseases is exposure to allergens, and existing research has demonstrated a dose–response relationship between the prevalence of allergic diseases and allergen specific IgE (sIgE) levels (10). This emphasizes the significance of recognizing various allergen types and their distribution patterns for effective prevention, diagnosis, and treatment. In clinical practice, the most prevalent allergen source testing methods are the skin prick test (SPT) and serum-sIgE assessments. Notably, serum sIgE testing stands out due to its characteristic of being unaffected by antihistamine drugs and avoidance of SPT-related discomfort, solidifying its reputation as a dependable approach for allergen source identification (11). The distribution of allergen sources is influenced by various factors, including ethnicity, climate, seasonal variations, and lifestyle (12–14). However, large-scale studies on allergen sources in patients with allergic diseases are scarce in Changzhou, eastern China. Therefore, in this study, we retrospectively analyzed the serum sIgE and total IgE results from patients with allergic diseases who were treated at Changzhou Third People's Hospital between January 2018 and December 2019, aiming to provide a basis for the precise diagnosis and treatment of allergic diseases in the eastern region of China.

## 2 Materials and methods

### 2.1 Study design

In this retrospective study, a total of 4,149 patients with suspected allergic diseases, including 2,229 males (53.72%) and 1,920 females (46.28%), were enrolled from January 2018 to December 2019 in Changzhou Third People's Hospital, Jiangsu Province, eastern China. All patients presented with allergic symptoms, such as nasal itching, sneezing, rhinorrhea, red or itchy eyes, skin itching, eczema, urticaria, coughing and shortness of breath. The age of the subjects ranged from 8 months to 91 years, and the subjects were categorized into nine groups: 1–5, 6–10, 11–15, 16–20, 21–30, 31–40, 41–50, 51–60, and >60 years. This stratification encompassed populations from childhood to the elderly. All participants were screened for serum sIgE and total IgE using the Mediwiss AllergyScreen system.

### 2.2 Allergyscreen test

Nineteen sIgE levels for inhaled and food allergen sources and total IgE levels were assessed in serum samples using the AllergyScreen test by Mediwiss Analytic GmbH, Moers, Germany, following the manufacturer's instructions. The inhaled allergen sources tested were *D. pteronyssinus*, house dust, mulberry, cat dander, dog hair, cockroache, amaranth, mold mix, grass mix, and tree pollen mix. Food allergen sources included egg, cow's milk, shrimp, beef, shellfish, crab, mango, cashew nut, and pineapple. The results were semi-quantitative and categorized based on the following scale: grade 0 (<0.35 IU/ml); grade I (0.35–0.70 IU/ml); grade II (0.71–3.5 IU/ml); grade III (3.6–17.5 IU/ml); grade IV (17.6–50 IU/ml); grade V (51–100 IU/ml); and grade VI (>100 IU/ml). A positive sIgE concentration was defined as ≥0.35 IU/ml. The total serum IgE levels were categorized into three groups: low-concentration group (<100 IU/ml); medium-concentration group (100–200 IU/ml); and high-concentration group (>200 IU/ml).

### 2.3 Sample size

The study was performed at the foremost allergic disease diagnosis and treatment center in Changzhou, where the sample size was set based on the number of patients who visited during the study period.

### 2.4 Statistical analysis

Data analysis was conducted using IBM SPSS Statistics for Windows, Version 27.0. The rates of sIgE positivity were compared using either the chi-square test or Fisher's exact probability method, whereas total IgE levels were compared using the Kruskal–Wallis test. Statistical significance was defined as *p* < 0.05.

## 3 Results

### 3.1 Characteristics of the study population

A total of 4,149 participants were enrolled in this retrospective study: 2,229 (53.72%) males and 1,920 (46.28%) females. The median age was 23 years (range: 8 months to 91 years). A total of 2,491 (60.04%) patients were positive for sIgE to at least one allergen source. A total of 997 participants (40.02%) were positive for one source and 1,494 (59.98%) were positive for two or more sources. The positive sIgE rate of inhaled allergen sources was 49.41% (2,050), significantly higher than that of food allergen sources 36.61% (1,519) (*χ*^2^ = 138.63, *p* < 0.001) (Table 1).

**Table 1:** Clinical characteristics of the patients.

Characteristics	Total, *N* = 4,149
Gender (*N*;%)	
Male	2,229 (53.72)
Female	1,920 (46.28)
Age (years)	
Median (IQR)	23 (9,28)
Range	0.75–91
Allergen sources sensitization^a^ (*N*; %)	
0	1,658 (39.96)
1	997 (24.03)
2	552 (13.30)
≥3	942 (22.70)
Inhaled	2,050 (49.41)
Food	1,519 (36.61)

^a^ Sensitization is defined as sIgE ≥0.35 IU/ml.

### 3.2 Prevalence of allergen source sensitization among patients

**Figure 1:**
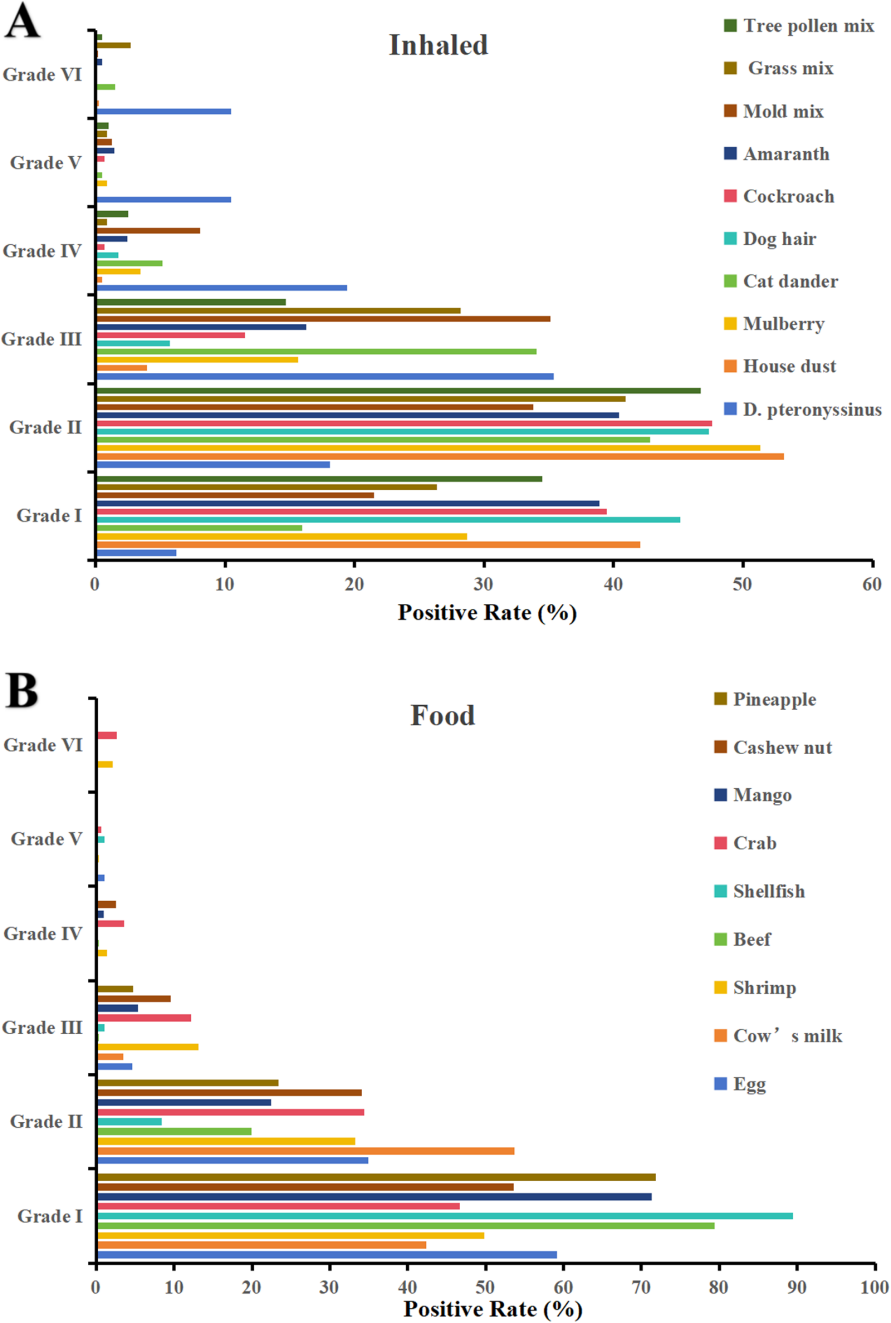
Distribution of sIgE grades for **(A)** inhaled and **(B)** food allergen sources (grade 0 excluded).

The most common inhaled allergen source was *D. pteronyssinus* (37.58%, 1,559/4,149), followed by mold mix (13.11%, 544/4,149) and house dust (9.11%, 378/4,149). The most common food allergen sources were cashew nut (15.57%, 646/4,149), cow's milk (10.99%, 456/4,149), and egg (9.33%, 387/4,149) (Table 2). As is shown in Figure 1, The majority of positive sIgE results were concentrated in the lower levels (grades I to III). *D. pteronyssinus* exhibited the most potent allergenicity, with 10.45% (163) of cases reaching grade VI, which was notably elevated compared with other allergens. Additionally, crab and shrimp were the only food allergen sources that elicited high-grade reactions, with eight and six cases of grade VI, respectively.

**Table 2:** Sensitization rates of 19 common allergen sources, *n* (%).

Allergen sources		Negative^a^ (Grade 0)	Positive^a^							
			Grade I	Grade II	Grade III	Grade IV	Grade V	Grade VI	Total	Ranking
Inhaled	D. *pteronyssinus*	2590 (62.4)	97	282	551	303	163	163	1559 (37.58)	1
	House dust	3771 (90.89)	159	201	15	2	0	1	378 (9.11)	6
	Mulberry	4034 (97.23)	33	59	18	4	1	0	115 (2.77)	16
	Cat dander	3955 (95.32)	31	83	66	10	1	3	194 (4.68)	14
	Dog hair	3923 (94.55)	102	107	13	4	0	0	226 (5.45)	10
	Cockroach	4002 (96.46)	58	70	17	1	1	0	147 (3.54)	15
	Amaranth	3946 (95.11)	79	82	33	5	3	1	203 (4.89)	12
	Mold mix	3605 (86.89)	117	184	191	44	7	1	544 (13.11)	3
	Grass mix	4039 (97.35)	29	45	31	1	1	3	110 (2.65)	17
	Tree pollen mix	3952 (95.25)	68	92	29	5	2	1	197 (4.75)	13
Food	Egg	3762 (90.67)	229	135	18	1	4	0	387 (9.33)	5
	Cow’s milk	3693 (89.01)	193	245	16	1	0	1	456 (10.99)	4
	Shrimp	3866 (93.18)	141	94	37	4	1	6	283 (6.82)	8
	Beef	3868 (93.23)	223	56	1	1	0	0	281 (6.77)	9
	Shellfish	4054 (97.71)	85	8	1	0	1	0	95 (2.29)	18
	Crab	3838 (92.50)	145	107	38	11	2	8	311 (7.50)	7
	Mango	3926 (94.63)	159	50	12	2	0	0	223 (5.37)	11
	Cashew nut	3503 (84.43)	346	220	62	16	1	1	646 (15.57)	2
	Pineapple	4085 (98.46)	46	15	3	0	0	0	64 (1.54)	19

^a^ grade 0 (< 0.35 IU/ml), Grade I (0.35–0.70 IU/mL), Grade II (0.71–3.5 IU/mL), Grade III (3.6–17.5 IU/mL), Grade IV (17.6–50 IU/mL), Grade V (51–100 IU/mL), Grade VI (>100 IU/mL).

### 3.3 Allergen source sensitization in different sex groups

The positive rates of sIgE in overall allergen sources (63.44% vs. 56.09%), inhaled allergen sources (53.30% vs. 44.90%), and food allergen sources (39.12% vs. 33.70%) in male patients were significantly higher than those in female patients (*χ*^2^ = 23.18; 29.13; 13.07, *p* < 0.001) (Table 3). Further analysis revealed that *D. pteronyssinus* (*χ*^2^ = 11.81, *p* < 0.001), house dust (*χ*^2^ = 6.70, *p* = 0.01), cat dander (*χ*^2^ = 16.78, *p* < 0.001), dog hair (*χ*^2^ = 31.00, *p* < 0.001), mold mix (*χ*^2^ = 11.49, *p* < 0.001), cow's milk (*χ*^2^ = 28.92, *p* < 0.001), shrimp (*χ*^2^ = 6.70, *p* = 0.01), beef (*χ*^2^ = 8.87, *p* = 0.003), crab (*χ*^2^ = 10.13, *p* = 0.001), and cashew nut (*χ*^2^ = 11.19, *p* < 0.001) showed the same trend (Table 4).

**Table 3:** Sensitization rates of overall allergen sources in different sex groups, *n* (%).

Allergen sources	Number of sIgE-positive patients		*χ* ^2^	*p*-value
	Male	Female		
Overall	1,414 (63.44)	1,077 (56.09)	23.18	<0.001
Inhaled	1,188 (53.30)	862 (44.90)	29.13	<0.001
Food	872 (39.12)	647 (33.70)	13.07	<0.001

**Table 4:** Sensitization rates of 19 allergen sources compared by sex, *n* (%).

Allergen sources	Number of sIgE-positive patients		*χ* ^2^	*p*-value
	Male	Female		
D. *pteronyssinus*	891 (39.97)	668 (34.79)	11.81	<0.001
House dust	227 (10.18)	151 (7.86)	6.70	0.01
Mulberry	68 (3.05)	47 (2.45)	1.39	0.24
Cat dander	132 (5.92)	62 (3.23%)	16.78	<0.001
Dog hair	162 (7.27)	64 (3.33)	31.00	<0.001
Cockroach	83 (3.72)	64 (3.33)	0.46	0.5
Amaranth	109 (4.89)	94 (4.90)	0.00	0.99
Mold mix	329 (14.76)	215 (11.20)	11.49	<0.001
Grass mix	62 (2.78)	48 (2.50)	0.32	0.57
Tree pollen mix	117 (5.25)	80 (4.17)	2.67	0.1
Egg	222 (9.96)	165 (8.60)	2.28	0.13
Cow's milk	299 (13.41)	157 (8.17)	28.92	<0.001
Shrimp	173 (7.76)	110 (5.73)	6.70	0.01
Beef	175 (7.85)	106 (5.52)	8.87	0.003
Shellfish	51 (2.29)	44 (2.29)	0.00	0.99
Crab	194 (8.70)	117 (6.09)	10.13	0.001
Mango	119 (5.34)	104 (5.42)	0.01	0.91
Cashew nut	386 (17.32)	260 (13.54)	11.19	<0.001
Pineapple	35 (1.57)	29 (1.51)	0.02	0.88

### 3.4 Allergen source sensitization in different age groups

**Figure 2:**
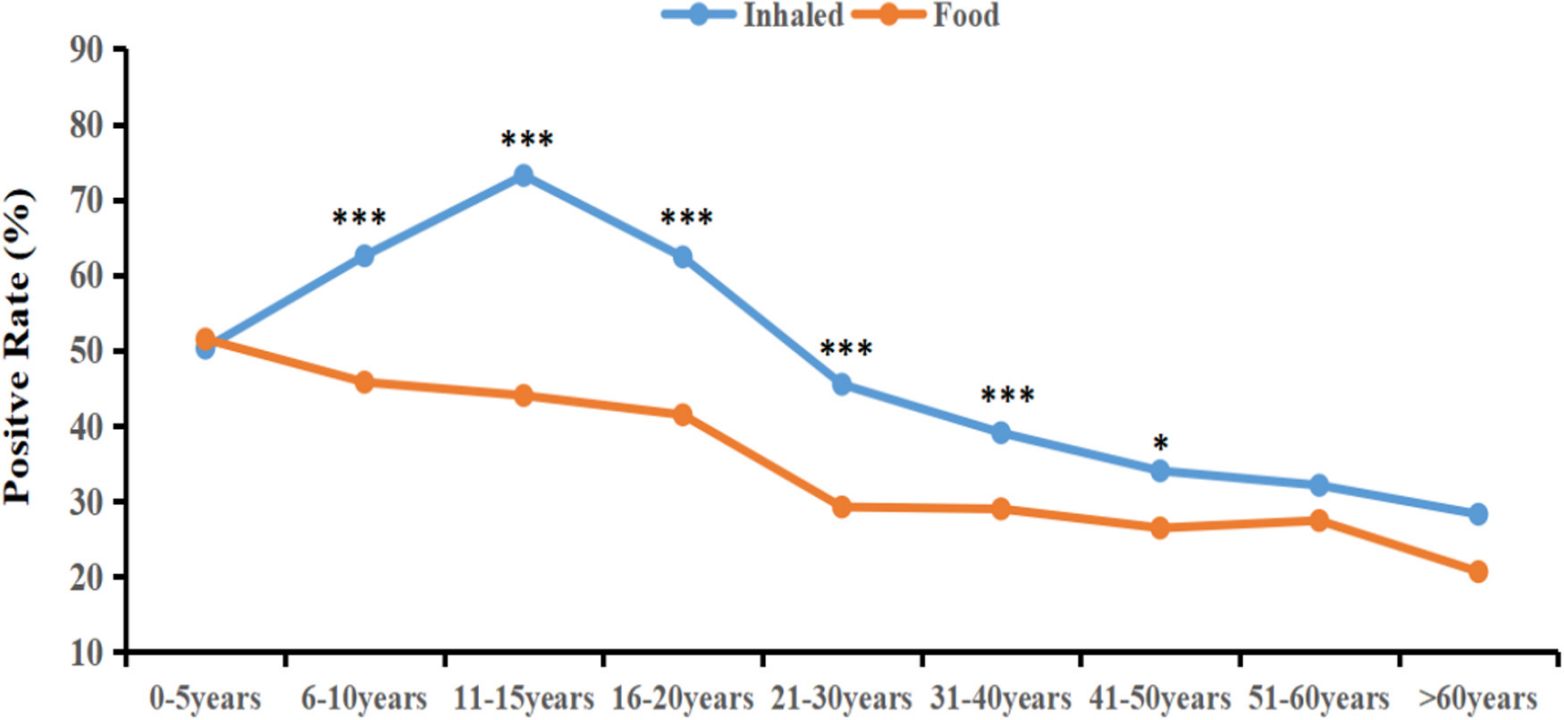
The sIgE-positive rates of inhaled and food allergen sources in differenr age groups. *P < 0.05; ***P < 0.001.

The sIgE-positive rates of inhaled allergen sources were significantly higher than those of food allergen sources ranged from 6 to 10 years to 41–45 years(*χ*^2^ = 47.61; 73.39; 20.55; 35.81; 13.72; 5.94, *p* < 0.05) (Table 5). As illustrated in Figure 2, the sensitization rate of inhaled allergen sources increased with age in the young age group, peaked at 11–15 years, and then declined. The sIgE-positive rates of food allergen sources decreased with increasing age. Table 6 shows that, except for mulberry, cockroach, tree pollen mix, shellfish, mango, and pineapple, the prevalence of sIgE for other inhalant and food allergen sources showed significant differences in different age groups (*p* < 0.05).

**Table 5:** Sensitization rates to inhaled and food allergen sources across nine age groups, *n* (%).

Allergen sources	Number of sIgE-positive patients								
	1–5 years	6–10 years	11–15 years	16–20 years	21–30 years	31–40 years	41–50 years	51–60 years	>60 years
*N*	501	841	418	234	633	604	435	299	184
Inhaled	252 (50.30)	526 (62.54)	306 (73.21)	146 (62.39)	288 (45.50)	236 (39.07)	148 (34.02)	96 (32.11)	52 (28.26)
Food	258 (51.50)	385 (45.78)	184 (44.02)	97 (41.45)	185 (29.23)	175 (28.97)	115 (26.44)	82 (27.42)	38 (20.65)
*χ* ^2^	0.14	47.61	73.39	20.55	35.81	13.72	5.94	1.57	2.88
*p*-value	0.71	<0.001	<0.001	<0.001	<0.001	<0.001	0.02	0.21	0.09

**Table 6:** Positive rates of sIgE to individual allergen sources in different age groups, *n* (%).

Allergen sources	Number of sIgE-positive patients									Chi square (*χ*^2^)	*p* value
	0–5 years	6–10 years	11–15 years	16–20 years	21–30 years	31–40 years	41–50 years	51–60 years	>60 years		
*N*	501	841	418	234	633	604	435	299	184		
D. pteronyssinus	148 (29.54)	433 (51.49)	267 (63.88)	125 (53.42)	231 (36.49)	179 (29.64)	98 (22.53)	53 (17.73)	25 (13.59)	385.38	<0.001
House dust	47 (9.38)	143 (17.00)	74 (17.70)	37 (15.81)	32 (5.06)	24 (3.97)	11 (2.53)	7 (2.34)	3 (1.63)	196.84	<0.001
Mulberry	6 (1.20)	17 (2.02)	11 (2.63)	8 (3.42)	16 (2.53)	19 (3.15)	21 (4.83)	11 (3.68)	6 (3.26)	15.11	0.06
Cat dander	60 (11.98)	59 (7.02)	26 (6.22)	13 (5.56)	18 (2.84)	11 (1.82)	5 (1.15)	2 (0.67)	0 (0)	120.62	<0.001
Dog hair	62 (12.38)	78 (9.27)	36 (8.61)	12 (5.13)	14 (2.21)	7 (1.16)	6 (1.38)	10 (3.34)	1 (0.54)	138.35	<0.01
Cockroach	8 (1.60)	25 (2.97)	14 (3.35)	11 (4.70)	23 (3.63)	24 (3.97)	18 (4.14)	17 (5.69)	7 (3.80)	12.16	0.14
Amaranth	8 (1.60)	34 (4.04)	11 (2.63)	12 (5.13)	33 (5.21)	41 (6.79)	35 (8.05)	19 (6.35)	10 (5.43)	33.21	<0.001
Mold mix	88 (17.56)	185 (22.00)	82 (19.62)	29 (12.39)	53 (8.37)	38 (6.29)	26 (5.98)	25 (8.36)	18 (9.78)	146.93	<0.001
Grass mix	4 (0.80)	17 (2.02)	9 (2.15)	4 (1.71)	16 (2.53)	23 (3.81)	19 (4.37)	11 (3.68)	7 (3.80)	20.35	0.01^a^
Tree pollen mix	18 (3.59)	39 (4.64)	21 (5.02)	12 (5.13)	27 (4.27)	26 (4.30)	26 (5.98)	20 (6.69)	8 (4.35)	6.24	0.62
Egg	114 (22.75)	125 (14.86)	46 (11.00)	17 (7.26)	31 (4.90)	22 (3.64)	17 (3.91)	10 (3.34)	5 (2.72)	214.87	<0.001
Cow's milk	139 (27.74)	174 (20.69)	68 (16.27)	18 (7.69)	23 (3.63)	12 (1.99)	11 (2.53)	6 (2.01)	5 (2.72)	393.59	<0.001
Shrimp	41 (8.18)	43 (5.11)	31 (7.42)	21 (8.97)	56 (8.85)	48 (7.95)	23 (5.29)	13 (4.35)	7 (3.80)	19.68	0.01
Beef	44 (8.78)	83 (9.87)	38 (9.09)	25 (10.68)	32 (5.06)	23 (3.81)	17 (3.91)	12 (4.01)	7 (3.80)	48.40	<0.001
Shellfish	18 (3.59)	20 (2.38)	7 (1.67)	7 (2.99)	16 (2.53)	10 (1.66)	7 (1.61)	10 (3.34)	0 (0)	13.82	0.08^a^
Crab	63 (12.57)	59 (7.02)	37 (8.85)	26 (11.11)	45 (7.11)	35 (5.79)	20 (4.60)	21 (7.02)	5 (2.72)	38.52	<0.001
Mango	26 (5.19)	45 (5.35)	17 (4.07)	21 (8.97)	30 (4.74)	28 (4.64)	29 (6.67)	21 (7.02)	6 (3.26)	13.20	0.11
Cashew nut	135 (26.95)	149 (17.72)	71 (16.99)	38 (16.24)	59 (9.32)	91(15.07)	48(11.03)	35(11.71)	20(10.87)	85.21	<0.001
Pineapple	6(1.20)	16(1.90)	3(0.72)	4(1.71)	7(1.11)	9(1.49)	4(0.92)	12(4.01)	3(1.63)	13.73	0.08^a^

^a^ Fisher's exact probability method.

As shown in Table 7, 1,157 patients (27.89%) were in the low-concentration group, 361 (8.70%) were in the medium-concentration group, and 2,631 (63.41%) were in the high-concentration group. The levels of total serum IgE varied across different age groups (H = 100.56; *p* < 0.001). The high-concentration group generally showed an increasing trend up to the age of 11–15 years, followed by a gradual decline.

**Table 7:** Total Serum IgE level in different age groups, *n* (%).

Total IgE	<100 IU∕ml	100–200 IU∕ml	>200 IU∕ml	H	*p*-value
*N*	1,157	361	2,631		
0–5 years	176 (35.13)	38 (7.58)	287 (57.29)	100.56	<0.001
6–10 years	211 (25.09)	55 (6.54)	575 (68.37)		
11–15 years	66 (15.79)	32 (7.66)	320 (76.56)		
16–20 years	43 (18.38)	14 (5.98)	177 (75.64)		
21–30 years	158 (24.96)	65 (10.27)	410 (64.77)		
31–40 years	179 (29.64)	76 (12.58)	349 (57.78)		
41–50 years	140 (32.18)	42 (9.66)	253 (58.16)		
51–60 years	116 (38.80)	22 (7.36)	161 (53.85)		
>60 years	68 (36.96)	17 (9.24)	99 (53.80)		

## 4 Discussion

Over the past few decades, the prevalence of allergic diseases has significantly increased worldwide, particularly in industrialized nations (15). A comprehensive study spanning 18 Chinese cities revealed that the self-reported prevalence of AR among adults was 17.6%, with asthma and atopic dermatitis affecting 4.2% and 2.42% of the adult population, respectively (16–18). It is worth noting that the incidence rates are even more pronounced among children and adolescents. Specifically, in Wuhan, China, 28.6% of children aged 6–12 years suffered from AR (19). Moreover, in 2020, the prevalence of asthma among 4-year-old urban boys in China escalated to 10.27%, and atopic dermatitis affected 12.94% of children aged 1–7 years (20, 21).

Previous research has highlighted substantial regional variations in allergen source distribution. In Europe and the Americas, common inhaled allergen sources include pollen, house dust mites, and pet dander, whereas food allergen sources predominantly consist of peanuts, milk, and eggs during childhood (22–25). Conversely, in Asia, sensitization to house dust mites is generally the most prevalent, but the spectrum of food allergies is more diverse (26–29). Notably, sensitization rates to shellfish and seafood in Southeast Asia are significantly higher than those in other regions (30). Furthermore, climatic conditions play a pivotal role in allergen source distribution. In tropical and subtropical regions, sensitization to house dust mites is often positively correlated with the annual average humidity, whereas in temperate regions, seasonal pollen allergies are more prevalent (31, 32).

Our study included 4,149 patients and revealed that 60.04% of the participants tested positive for sIgE from at least one allergen source. Among them, 59.98% were positive for two or more allergen sources. Identifying the primary causative allergen source is essential among polysensitized individuals, as allergen avoidance is a key therapeutic approach for allergic diseases. Therefore, it is vital to combine clinical data, patient history, and allergen challenge tests to precisely identify the main culpable allergen source, thereby guiding future medical practices. We found the sIgE-positive rate of inhaled allergen sources (49.41%) to be significantly higher than that of food allergen sources (36.61%, *P* < 0.05), which is similar to the findings of previous studies (33, 34). Among the inhaled allergen sources, *D. pteronyssinus* emerged as the most prevalent (37.58%), echoing broader trends in Asia (35, 36). Notably, mold mix (13.11%) and house dust (9.11%) also exhibited relatively high positive rates of sIgE. This could be linked to Changzhou's subtropical maritime climate, which is characterized by persistent high humidity and rainfall, especially during the plum rain season, creating an ideal habitat for mold and mite growth. Simultaneously, owing to the relatively developed economy of Changzhou and the escalating trend of pet ownership among households, we noted substantial positive sIgE rates of 5.45% for dog hair and 4.68% for cat dander. These findings indicate the necessity for heightened vigilance toward pet allergen prevention in the future. In terms of food allergen sources, cashew nut was the predominant source (15.57%), followed by cow's milk (10.99%). This result is similar to Sun's findings (37), potentially attributed to regional dietary disparities. For instance, the prevalent consumption of snacks made from nuts and the increased intake of dairy products could influence these patterns (38, 39). In this study, *D. pteronyssinus* not only topped the allergen source-positive rate but also exhibited a significantly higher proportion of high-grade sensitization compared with other allergen sources. This may stem from prolonged exposure coupled with intense immune responses. Clinicians should remain vigilant regarding severe allergic reactions, which can escalate rapidly and pose fatal risks. A previous study indicated that sIgE activity can affect the severity of allergic reactions (40)^.^ It is well known that dust mites and shrimp share cross-allergenic reactions via tropomyosin (41). Furthermore, due to the coastal geographical location and abundant seafood supply, seafood has become a primary food allergen source in China, Thailand, Singapore, and other Asian countries (30). Our findings highlighted eight crab and six shrimp cases with sIgE levels equivalent to grade VI, suggesting that individuals who are allergic to crab or shrimp should be particularly cautious about severe allergic reactions.

Previous research has revealed sex differences in allergen source sensitization rates, with some studies indicating higher rates among males and others reporting the opposite among females (42, 43). We found that male patients exhibited elevated sensitization rates for overall allergen sources (63.44% compared to 56.09% in females), inhaled allergen sources (53.30% vs. 44.90%), and food allergen sources (39.12% vs. 33.70%) (*P* < 0.001). This observed sex disparity could be attributed to factors such as increased outdoor activity or occupational exposure among males, or the influence of male hormones on immune system responses, as suggested by previous studies (44). However, there is currently a dearth of direct evidence to corroborate these theories. Future investigations should explore sex-specific sensitization pathways through cohort studies or by examining molecular mechanisms.

The distribution of allergen sources among patients with allergic diseases varies according to age. Epidemiological studies have revealed that infants mostly react positively to food allergen sources, whereas school-aged children are more sensitive to inhaled allergen sources (45–47). Our study indicates that the highest positive rate of sIgE for inhaled allergen sources was observed during adolescence (11–15 years), reaching 73.21%, and then decreased with advancing age. Conversely, the positive rate of food allergen sources decreased steadily with age. Notably, from age 6–50 years, the positive rate for inhaled allergen sources surpassed that of food allergen sources, which is consistent with the aforementioned trend. This trend could be attributed to factors such as increased indoor activity and the immune system's maturation. Infants aged 0–5 years exhibited significantly higher positive rates for milk, eggs, and cashews than other age groups. This may be explained by their underdeveloped intestinal barrier and incomplete immune tolerance. Furthermore, the sensitization rates to cat and dog dander were significantly higher in this age group, reaching 11.98% and 12.38%, respectively. This could be due to their immature immune systems and possibly greater exposure to pets in households in the studied region. Moreover, Zhu et al. found that among children sensitized to cat or dog allergen sources, the sensitization rate was significantly higher in patients with a parental allergy history than in those without a family history of allergic diseases (48). Therefore, parents are advised to minimize close contact between infants and pets.

The distribution of total serum IgE levels showed that the high-concentration IgE group (>200 IU/ml) accounted for the largest proportion (63.41%), peaking at 76.56% in the 11–15 years group. This finding is consistent with active hormonal changes and immune responses during adolescence (49). With increasing age, the proportion of patients in the high-concentration IgE group gradually decreased, possibly due to immunosenescence or changes in chronic inflammatory states (50).

This study has several limitations. First, due to its retrospective cross-sectional design, we were unable to ascertain causal connections between allergen source exposure and sensitization, thereby necessitating longitudinal studies for further verification. Second, the insufficient examination of the molecular mechanisms underlying sex disparities and the elevated rate of allergen source sensitization requires additional experimental research. Third, the lack of environmental data, including *D. pteronyssinus* concentrations and dietary habits, may lead to biases in the interpretation of the results. Fourth, multiple studies have revealed that the positive rate of SPT is higher than that of sIgE detection (51, 52). However, in the current investigation, SPT outcomes were not considered. Forthcoming research could greatly benefit from an integrative examination that merges both sIgE and SPT information, ultimately improving the precision of allergen source diagnosis. Lastly, the AllergyScreen panel used in this study did not include some predefined allergen sources such as fish. In future studies, we will make sure to incorporate fish allergens into the testing panel to provide a more comprehensive evaluation of seafood-related sensitization patterns.

## 5 Conclusion

In this study, we comprehensively determined the allergen source sensitization profiles of patients with allergic diseases in Changzhou. We elucidated the central role of *D. pteronyssinus* and the influence of age and sex on allergy patterns. Notably, adolescents (11–15 years) exhibited the highest sensitization rates to inhaled allergen sources, potentially due to immune system maturity and increased environmental exposure. Conversely, the prevalence of food allergen sources decreased with age, possibly indicating the development of oral tolerance. These findings provide a scientific basis for crafting tailored regional and age-tailored strategies for allergy prevention and management, such as mite control in educational institutions and early dietary guidance for infants.

## Data Availability

The raw data supporting the conclusions of this article will be made available by the authors, without undue reservation.
